# Manifestations and management of Sjögren’s disease

**DOI:** 10.1186/s13075-024-03262-4

**Published:** 2024-02-08

**Authors:** Mehrnaz Maleki-Fischbach, Liudmila Kastsianok, Matthew Koslow, Edward D. Chan

**Affiliations:** 1 Division of Rheumatology and Department of Medicine, National Jewish Health, 1400 Jackson Street, Denver, CO 80206 USA; 2 Division of Pulmonary, Critical Care and Sleep Medicine, National Jewish Health, Denver, CO USA; 3 Pulmonary Section, Rocky Mountain Regional Veterans Affairs Medical Center Aurora, Aurora, CO USA

**Keywords:** Sjögren’s syndrome, Pulmonary fibrosis, Systemic manifestations, Treatment

## Abstract

Sjögren’s disease is a heterogeneous autoimmune disorder that may be associated with systemic manifestations such as pulmonary or articular involvement. Systemic complications have prognostic implications and need to be identified and managed in a timely manner. Treatment should be tailored to the type and severity of organ involvement, ideally based on multidisciplinary evaluation.

## Introduction

Sjögren’s disease is a systemic autoimmune disorder characterized by hypofunction of salivary and lacrimal glands, which may have protean manifestations [1]. Historically, Sjögren’s disease was referred to as primary when the clinical manifestations occurred alone and as secondary when they were associated with another systemic autoimmune disease; however, primary Sjögren’s disease is now commonly referred to as Sjögren’s disease and secondary Sjögren’s disease as Sjögren’s disease with overlap. The estimated incidence and prevalence of Sjögren’s disease vary depending on the classification criteria used. In a systematic review of published literature, the incidence of Sjögren’s disease was estimated to be 6.9 per 100,000 person-years and the prevalence to be 60.8 cases per 100,000 [2]. This disease has a strong female predominance, with a reported female-to-male ratio ranging from 9:1 to 28:1 [2, 3]. The typical age of onset is in the fourth or fifth decade [2, 4]. The prevalence of secondary Sjögren’s disease (Sjögren’s disease with overlap) varies depending on the associated autoimmune disease. Among 300 patients at a tertiary care center, the prevalence of Sjögren’s disease with overlap was estimated to be 15% in patients with systemic lupus erythematosus, 20% in patients with rheumatoid arthritis, and 30% in patients with systemic sclerosis [5].

Sjögren’s disease tends to be a slowly progressive disease [6], but patients may develop systemic manifestations such as interstitial lung disease (ILD) and complications such as lymphoma, which significantly impact prognosis [6–8]. In this article, we review the clinical manifestations of Sjögren’s disease and their implications for prognosis and management.

### Pathogenesis

The pathogenesis of Sjögren’s disease has not been fully elucidated but is likely a multifactorial process involving genetic susceptibility and environmental triggers that lead to an abnormal immune response [9, 10]. Most of the genes that have been identified as being associated with Sjögren’s disease are related to alterations in immune function, particularly innate immunity and inflammatory signaling [11–13]. The most robust association has been identified for HLA class II genes, such as *HLA-DQB1* and *HLA-DQA1* [14], reflecting that antigen presentation to CD4 + T cells, which leads to excessive immune activation, is an important pathogenic mechanism of the disease. In addition, several genes associated with Sjögren’s disease are involved in B cell function [11], supporting the role of dysregulated adaptive immune responses in the disease process. Epigenetic processes such as DNA methylation have also been implicated [15, 16], as have non-coding RNAs, of which microRNAs have been the most extensively studied [17, 18].

It is hypothesized that, in the initiation step, environmental triggers such as viral infection, combined with genetic predisposition and epigenetic factors, disrupt the salivary gland epithelium [9, 10, 19]. Increased levels of cytokines, including type I interferon and B cell activating factors, and chemokines promote the migration of lymphocytes and dendritic cells, resulting in chronic inflammation of the exocrine glands [10, 20, 21]. In this inflammatory microenvironment, antigen-presenting cells process and present viral and auto-antigens, leading to the activation of autoreactive T and B cells [10]. These events induce tissue damage via the secretion of cytotoxic granules, further disrupting the epithelium and amplifying exposure to autoantigens [9]. Immune complexes that form between autoantibodies and autoantigens bind to receptors on dendritic cells, augmenting type I interferon production and creating a self-perpetuating cycle of epithelial and immune cell interaction and autoimmunity [9].

### Classification criteria

The hyperactivation of B lymphocytes in patients with Sjögren’s disease results in the production of many circulating autoantibodies. Traditional biomarkers include anti-Sjögren’s disease-related antigens A and B (anti-SSA/Ro and anti-SS-B/La) antibodies, antinuclear antibody (ANA), and rheumatoid factor (RF). Anti-SSA/Ro antibodies are present in nearly 75% of patients with Sjögren’s disease [22] and play a key role in diagnosis [1]. Anti-Ro antibodies, detected by a solid phase immunoassay that includes a mixture of Ro60 and Ro52, are relatively specific for Sjögren’s disease [23].

The classification criteria for Sjögren’s disease have been developed by expert working groups [1, 24, 25]. These were originally developed as criteria for enrolling patients into clinical trials but can be used as a guide for diagnosis. Based on the American College of Rheumatology (ACR)/European Alliance of Associations for Rheumatology (EULAR) consensus classification criteria developed in 2017, individuals with signs or symptoms of Sjögren’s disease are classified as having Sjögren’s disease based on a scoring system that incorporates anti-SSA/Ro antibody positivity, focal lymphocytic sialadenitis focus score, ocular staining score, Schirmer’s test, and unstimulated salivary flow rate [1]. RF and anti-SS-B/La were not included in these criteria but are found in nearly 50% of patients [22].

### Symptoms

Sjögren’s disease has heterogeneous clinical manifestations. Dryness of the eyes (xerophthalmia) and mouth (xerostomia) is present in almost all patients. Data from over 6000 patients in the Big Data Sjögren Project Consortium registry found that 92% of patients had dry eyes and 94% had dry mouth [26]. General manifestations such as fatigue [27, 28], sleep disturbance [29, 30], and widespread pain [31] are also common. In a single-center study of 50 patients, fatigue and generalized body pain in the past 3 months were reported by 88% and 80% of patients, respectively [31].

### Prevalence of systemic manifestations

**Fig. 1:**
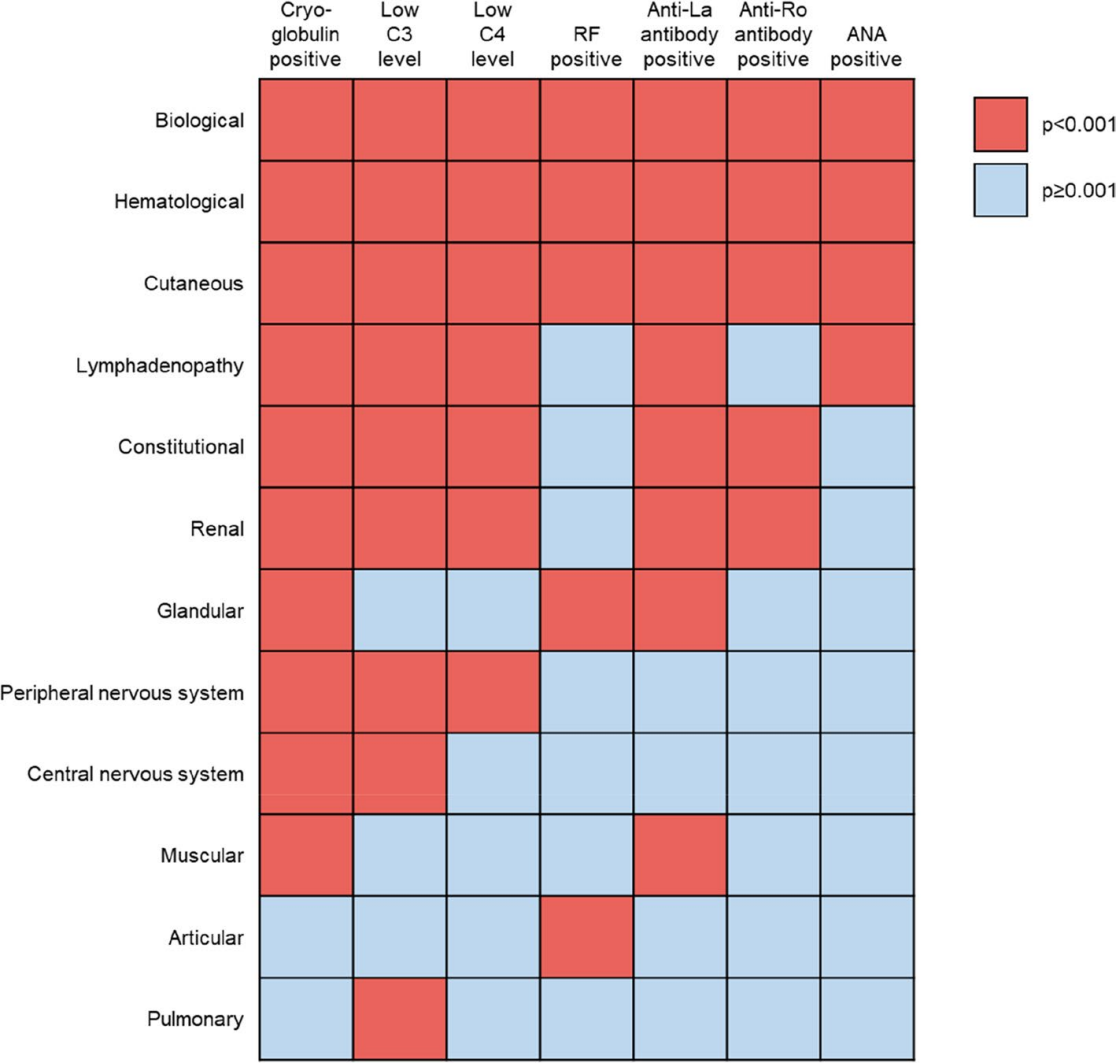
Associations between immunological markers and disease phenotype based on ESSDAI domains. Heat map of the main associations between immunological markers and disease phenotype based on ESSDAI domains in patients with Sjögren’s disease in the Big Data Sjögren Project [Adapted from 22]. ESSDAI, EULAR Sjögren’s Syndrome Disease Activity Index; C3, complement component C3; C4, complement component C4; RF, rheumatoid factor; ANA, antinuclear antibodies

Systemic manifestations of Sjögren’s disease are common. In a multi-center study of 395 patients with Sjögren’s disease, 30% had systemic manifestations at the time and 39% had experienced them in the past [32]. In another study of 1115 patients, 15% of the patients had severe extra-glandular manifestations treated with immunosuppressive drugs [33]. The EULAR Sjögren’s Syndrome Disease Activity Index (ESSDAI) score classifies systemic disease activity from low to high based on 12 domains (cutaneous, renal, articular, muscular, peripheral nervous system, central nervous system, hematological, glandular, constitutional, lymphadenopathic, pulmonary, biological) [34]. Among 921 patients in a Spanish registry, only 8% of patients with Sjögren’s disease had no systemic disease activity based on ESSDAI score over a mean follow-up of 6 years [35]. Excluding the hematological and biological domains, the most common ESSDAI domains with any level of activity were the articular (56%), glandular (34%), pulmonary (15%), and cutaneous (13%) domains [35]. Activities in the peripheral nervous system domain and central nervous system domain were reported in 10% and 3% of patients, respectively [35]. Patients with Sjögren’s disease may also develop systemic manifestations that are not included in the ESSDAI such as cardiovascular manifestations and Raynaud’s phenomenon [26]. Systemic involvement may be related to immunological dysregulation. Analyses of the Big Data Sjögren Project Consortium registry found that hypocomplementemia and cryoglobulinemia correlated with high systemic activity based on ESSDAI score and had a greater influence on phenotype than the presence of Ro/La autoantibodies and ANA (Fig. 1) [22].

### Lymphoma

Patients with Sjögren’s disease are at an increased risk of B cell lymphoma. Recent studies suggest that patients with primary Sjögren’s disease have a four- to sevenfold higher risk of non-Hodgkin’s lymphoma compared with the general population [36–38]. Non-Hodgkin’s lymphoma occurs in approximately 2–9% of patients with primary Sjögren’s disease [39, 40], with the mucosa-associated lymphoid tissue (MALT) subtype accounting for approximately two-thirds of cases [39].

### Articular involvement

A retrospective study of 419 patients with primary Sjögren’s disease found a prevalence of articular manifestations (arthralgia or non-erosive arthritis) of 45% [41]. Among 921 patients in a Spanish registry, 56% had articular involvement based on the ESSDAI domain [35]. A systematic review based on data from 152 patients found that arthritis was predominantly reported in the proximal interphalangeal joint (35%), metacarpophalangeal joint (35%), and wrist (30%) [42].

### Pulmonary involvement

Cough and dyspnea are common among patients with Sjogren’s disease [43]. Pulmonary disease may be diagnosed before, at the same time, or after a diagnosis of Sjögren’s disease is made [44–46]. Patients may initially present with unexplained cough due to airway disease, interstitial lung disease, or both, following which elicitation of sicca symptoms reveals undiagnosed Sjogren’s disease [45]. Several observational studies have demonstrated that respiratory symptoms, abnormal imaging findings, and impaired pulmonary physiology are often reported among patients with Sjögren’s disease [3, 47, 48]. In a US population-based cohort, the cumulative incidence of ILD was estimated as 10% 1 year after diagnosis and 20% 5 years after diagnosis of primary Sjögren’s disease [49]. The risk of developing ILD is higher when the anti-SSA/Ro-52 antibody is present [50].

Lung involvement may include parenchymal findings (non-specific interstitial pneumonia, usual interstitial pneumonia, lymphocytic interstitial pneumonia, and organizing pneumonia) and airway disease due to mucosal dryness (bronchitis sicca), lymphocytic infiltration such as follicular bronchiolitis and lymphocytic bronchitis, and bronchiectasis (due to chronic inflammation and inspissated mucus) [51, 52]. Airflow limitation due to these Sjögren’s-associated airway diseases, especially with significant reversibility with bronchodilators and/or glucocorticoids, may resemble asthma [51]. Superimposed lung disease due to non-tuberculous mycobacteria is not uncommon [53]. ILD may lead to pulmonary fibrosis. Among 34 patients with Sjögren’s disease and ILD at an Italian center, a fibrotic pattern on high-resolution computed tomography (HRCT) was detected in 53% of patients [54]. Among 151 patients with Sjögren’s disease and ILD at a center in China, a fibrotic pattern on HRCT was detected in 32% of patients [55].

### Cutaneous involvement

Among 921 patients with Sjögren’s disease in a Spanish registry, 13% had cutaneous involvement based on activity in the ESSDAI domain [35]. Cutaneous vasculitis is the most common type of dermatological involvement, characterized by palpable purpura [42, 56, 57]. Other cutaneous manifestations include annular erythema (or subacute cutaneous lupus erythematosus), erythema nodosum, and livedo reticularis [42, 56].

### Neurological involvement

The prevalence of peripheral nervous system involvement in patients with primary Sjögren’s disease has been reported as between 2 and 17%, with pure sensory neuropathies and axonal sensorimotor polyneuropathies the most common manifestations [58, 59]. Small-fiber neuropathy has been reported in 24–55% of cases of sensory neuropathy [58, 60]. In an analysis of 921 patients in a Spanish registry, 3% of patients had central nervous system involvement based on activity in the ESSDAI domain [35]. Among 424 patients with primary Sjögren’s disease at an Italian center, 6% had central nervous system involvement, with the most common manifestations being diffuse involvement, focal or multifocal lesions, multiple sclerosis-like disease, and isolated optic neuritis [61]. Factors associated with central nervous system involvement included disease duration, lung involvement, and decreased C4 levels. Dysfunction of the autonomic nervous system may also occur. In a prospective study of 317 patients with Sjögren’s disease, 55% had autonomic dysfunction based on the Composite Autonomic Symptom Scale (COMPASS) [62].

### Renal involvement

Renal involvement is reported in up to 10% of patients with Sjögren’s disease [35, 42, 63, 64]. It is usually characterized by tubulointerstitial nephritis with or without tubular acidosis and glomerulonephritis [64, 65].

### Impact of systemic manifestations on prognosis

Sjögren’s disease often follows a largely benign course, but patients’ prognosis can be markedly worsened by systemic manifestations. A systematic review that analyzed data from 14 studies found a 1.5-fold increase in mortality in patients with Sjögren’s disease compared with the general population [8]. Vasculitis, ILD, hypocomplementemia, and cryoglobulinemia were associated with a significantly increased risk of mortality, as were positivity for anti-La/SSB, older age, and male sex [8]. Among 1580 patients in a Spanish registry, 13% were classified as presenting with a life-threatening systemic disease, defined as high systemic ESSDAI activity in at least one organ domain [66]. The most common of these presentations were lymphoma, neurological involvement, and pulmonary involvement. Mortality over 10 years was 33% in patients with high activity in more than one organ domain compared to 20% in the overall cohort [66].

Lymphoma is one of the leading causes of death in patients with Sjögren’s disease [6]. In a retrospective study of 723 patients, 20% of deaths were caused by lymphoma [66]. Glomerulonephritis in patients with Sjögren’s disease is associated with an increased risk of lymphoma and mortality due to lymphoma [63]. Purpura may be associated with cryoglobulinemic vasculitis, a systemic vasculitis with complement activation that is linked to an increased risk of lymphoma and death [26, 57].

Pulmonary involvement also impacts survival. A US population-based cohort found that the development of ILD in patients with Sjögren’s disease was associated with poorer survival, with a hazard ratio of 2.16 over 9 years of follow-up [49]. Data from 216 patients in a Norwegian registry showed that those with pulmonary symptoms and either abnormal findings on HRCT or impaired pulmonary function tests (PFTs) had a fourfold increased risk of mortality in the 10 years following diagnosis of Sjögren’s disease than those without pulmonary involvement [47].

Measurement of systemic activity using the ESSDAI may help to identify patients with a poor prognosis who require more intensive monitoring and treatment. Among 1045 patients with Sjögren’s disease, high activity (score of 3) in at least one ESSDAI domain, an ESSDAI score ≥ 14, and the presence of more than one laboratory marker (lymphopenia, anti-SSB/La antibody, monoclonal gammopathy, hypocomplementemia, or cryoglobulinemia) were associated with an increased risk of mortality (hazard ratios of 2.14, 1.85, and 2.82, respectively) [7].

### Management of Sjögren’s disease

The management of patients with Sjögren’s disease should be based on a multidisciplinary evaluation of symptoms and systemic manifestations. Recommendations published by EULAR provide a framework for the use of therapies to treat symptoms of dryness, such as muscarinic agonists, saliva substitutes, and ocular tears, and for the use of immunosuppressive agents to treat systemic manifestations (Table 1) [67]. It is recommended that systemic immunomodulatory therapies be reserved for patients with active systemic disease, defined as a clinical ESSDAI score ≥ 1. Glucocorticoids should be used at the minimum dose and for the shortest time necessary to control systemic disease. Immunosuppressive agents such as leflunomide, methotrexate, azathioprine, mycophenolate, or cyclophosphamide, and biologics such as rituximab or tocilizumab are second/third-line options for patients who are intolerant or refractory to glucocorticoids have severe disease, or for whom long-term glucocorticoid use is anticipated [67, 68]. In randomized placebo-controlled trials, the anti-tumor necrosis factor (TNF) agents infliximab and etanercept failed to demonstrate efficacy as a treatment for Sjögren’s disease [69, 70].

**Table 1:** European Alliance of Associations for Rheumatology (EULAR) recommendations for the management of primary Sjögren’s disease [67]

Oral and ocular dryness punctum plugs	• The first therapeutic approach for oral dryness according to salivary gland function may be: – Non-pharmacological stimulation for mild dysfunction – Pharmacological stimulation with muscarinic agonists (e.g., pilocarpine, cevimeline) for moderate dysfunction – Saliva substitution for severe dysfunction • The first-line therapeutic approach to ocular dryness includes the use of artificial tears and ocular gels or ointments. Refractory or severe ocular dryness may be managed using topical immunosuppressive-containing drops and autologous serum eye drops
Fatigue and pain	• The severity of fatigue and pain should be scored using specific tools. Concomitant diseases should be evaluated • Analgesics or other pain-modifying agents should be considered for musculoskeletal pain
Systemic disease manifestations	• Treatment of systemic disease should be tailored to organ-specific severity using the EULAR Sjögren’s Syndrome Disease Activity Index definitions • Glucocorticoids should be used at the minimum dose and length of time necessary to control systemic disease • Immunosuppressive agents should mainly be used as glucocorticoid-sparing agents • B cell-targeted therapies may be considered in patients with severe refractory systemic disease • The organ-specific therapeutic approach may follow, as a general rule, the sequential (or combined) use of glucocorticoids, immunosuppressive agents, and biologic agents
B cell lymphoma	• Treatment of B cell lymphoma should be individualized according to histological subtype and disease stage

The lack of robust evidence to support the efficacy of treatments in Sjögren’s disease means that management is generally based on extrapolation of observations from other autoimmune diseases. To date, the only therapies to have met their primary outcome of improvement in ESSDAI score in randomized placebo-controlled trials in patients with Sjögren’s disease are leflunomide in combination with hydroxychloroquine [71], iscalimab (anti-CD40 monoclonal antibody) [72], ianalumab (anti-B cell-activating factor receptor antibody) [73] and low-dose interleukin 2 [74]. Treatment guidelines for rheumatologic manifestations of Sjögren’s disease issued by the Sjögren’s Syndrome Foundation in 2017 provided a moderately strong recommendation for the use of rituximab (anti-CD20 antibody) in patients with vasculitis, cryoglobulinemia associated with vasculitis, severe parotid swelling, inflammatory arthritis, pulmonary disease, and/or peripheral neuropathy, based on data from non-randomized studies [75]. Recently, a randomized placebo-controlled trial showed that the combination of rituximab and belimumab (anti-B cell-activating factor antibody) was well tolerated (primary outcome) and improved ESSDAI score in patients with Sjögren’s disease [76].

The failure of many clinical trials to show the benefit of therapy likely reflects the heterogeneity of Sjögren’s disease. There is growing interest in the identification of subgroups of patients who have different treatment responses [77–79]. For example, analyses of four subgroups based on symptoms in the UK Primary Sjögren’s Syndrome Registry indicated that the effects of rituximab and hydroxychloroquine on the EULAR Sjögren’s Syndrome Patient Reported Index may differ across subgroups [77, 78].

**Fig. 2:**
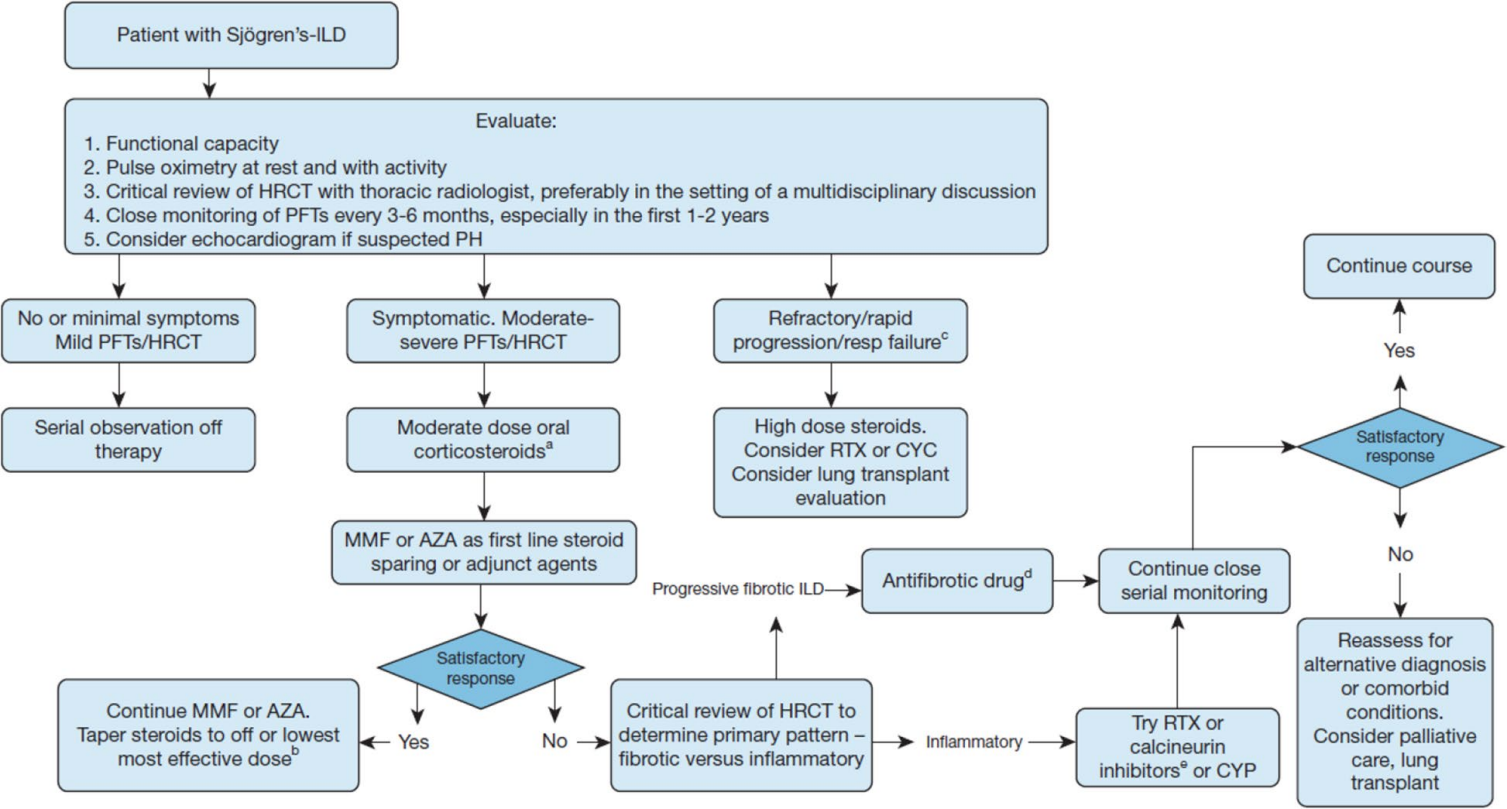
Evaluation and management of patients with Sjögren’s disease and symptoms/signs of interstitial lung disease. Recommendations for evaluation and management of patients with Sjögren’s disease and symptoms and/or signs of interstitial lung disease developed by the Sjögren’s Foundation [81]. ^a^Dose and duration of corticosteroids in Sjögren’s-ILD are not standardized. The panel proposed ≤ 60 mg daily of prednisone with a slow taper over weeks/months. In rapidly progressive ILD or acute respiratory failure, pulse-dose IV corticosteroids or high-dose oral corticosteroids up to 60 mg daily of prednisone should be considered. ^b^Steroid-sparing agents should be initiated as maintenance therapy in patients who are not able to taper off corticosteroids or who experience adverse effects or if long-term corticosteroid therapy is predicted. ^c^Condition rapidly deteriorates and requires hospitalization. ^d^Nintedanib is approved by the US Food and Drug Administration for the treatment of progressive fibrotic lung disease. ^e^Calcineurin inhibitors can be considered in patients who are intolerant to the initial maintenance therapy; there is no evidence to support superiority in patients who fail first-line therapy. AZA, azathioprine; CYC, cyclophosphamide; CYP, cyclosporine; HRCT, high-resolution computed tomography; ILD, interstitial lung disease; MMF, mycophenolate mofetil; PFTs, pulmonary function tests; PH, pulmonary hypertension; RTX, rituximab. Reprinted from Chest, Vol 159, Lee et al., Consensus guidelines for evaluation and management of pulmonary disease in Sjögren’s, pages no. 16, Copyright 2021, with permission from Elsevier

The prognostic implications of serious systemic complications make it paramount that these are identified and treated in a timely manner. Treatment should be tailored to the type and severity of organ involvement (Table 1) [67]. For patients with lymphoma, therapy should be individualized according to histological subtype [80], with an individualized approach directed by an oncologist. For patients with ILD, the treatment approach should be based on the severity of symptoms, level of physiologic impairment, and extent of radiographic disease. There is no evidence from controlled trials to support the use of glucocorticoids or immunosuppressants to treat ILD associated with Sjögren’s disease, but prednisone is considered an initial treatment option, with a steroid-sparing agent such as azathioprine or mycophenolate added if an initial response is seen. In 2021, the Sjögren’s Foundation published clinical practice guidelines for the evaluation and management of pulmonary involvement [81]. These recommended that therapeutic decisions for patients with pulmonary manifestations should ideally be based on multidisciplinary discussion and should be individualized based on the type of pulmonary involvement, PFTs, and findings on HRCT. Recommendations for the management of ILD were based on the severity according to the pulmonary domain of the ESSDAI, which is based on symptoms defined using the New York Heart Association (NYHA) Functional Classification, imaging, and PFTs. It was recommended that patients with ILD who have no or minimal respiratory symptoms and mild impairment on PFTs/HRCT can be closely monitored without treatment, while those with rapid deterioration may require pharmacological therapy or, if refractory to treatment, referral for lung transplant (Fig. 2) [81]. A guideline recently issued by the ACR included conditional recommendations for first-line treatment of ILD in patients with Sjögren’s disease with glucocorticoids, mycophenolate, azathioprine, rituximab, and cyclophosphamide [82].

The tyrosine kinase inhibitor nintedanib has been licensed for the treatment of progressive pulmonary fibrosis of any etiology and was recommended for use in patients with progressive pulmonary fibrosis in a clinical practice guideline published by international respiratory societies [83]. A randomized placebo-controlled trial conducted in patients with progressive fibrosing ILDs other than idiopathic pulmonary fibrosis showed that nintedanib slowed the rate of decline in forced vital capacity, with no heterogeneity in its treatment effect detected among subgroups by diagnosis [84–86].

Patients with fatigue and sleep disturbance may benefit from non-pharmacological therapies such as exercise [87, 88] or cognitive behavioral therapy [89]. Patient empowerment and access to social support are important to improve patients’ participation in activities of daily living [90].

## Conclusions

Sjögren’s disease is a heterogeneous disease that may be associated with systemic complications that impact prognosis. Understanding of the characteristics associated with different clinical and immunologic expressions of the disease has advanced in recent years, but further research is needed. The management of patients with Sjögren’s disease should be based on a multidisciplinary and individualized approach depending on the type and severity of organ involvement.

## Data Availability

Not applicable.

## Abbreviations

ACR: American College of Rheumatology
ANA: Antinuclear antibody
ESSDAI: EULAR Sjögren’s Syndrome Disease Activity Index
EULAR: European Alliance of Associations for Rheumatology
HRCT: High-resolution computed tomography
ILD: Interstitial lung disease
MALT: Mucosa-associated lymphoid tissue
NYHA: New York Heart Association
PFT: Pulmonary function test
RF: Rheumatoid factor
TNF: Tumor necrosis factor
